# Establishment of tumor microenvironment-preserving organoid model from patients with intracranial meningioma

**DOI:** 10.1186/s12935-024-03225-4

**Published:** 2024-01-18

**Authors:** Dokyeong Kim, Junseong Park, Hyeon-Chun Park, Songzi Zhang, Minyoung Park, Soon A. Park, Sug Hyung Lee, Youn Soo Lee, Jae-Sung Park, Sin-Soo Jeun, Yeun-Jun Chung, Stephen Ahn

**Affiliations:** 1 Precision Medicine Research Center, College of Medicine, The Catholic University of Korea, Seoul, Republic of Korea; 2 Department of Biomedicine and Health Sciences, College of Medicine, The Catholic University of Korea, Seoul, Republic of Korea; 3 Cancer Evolution Research Center, College of Medicine, The Catholic University of Korea, Seoul, Republic of Korea; 4 Department of Microbiology, College of Medicine, The Catholic University of Korea, 222 Banpo-daero, Seocho-gu, Seoul, 06591 Republic of Korea; 5 Department of Neurosurgery, Seoul St. Mary’s Hospital, College of Medicine, The Catholic University of Korea, 222 Banpo-daero, Seocho-gu, Seoul, 06591 Republic of Korea; 6 Department of Pathology, College of Medicine, The Catholic University of Korea, Seoul, Republic of Korea; 7 Department of Hospital Pathology, Seoul St. Mary’s Hospital, College of Medicine, The Catholic University of Korea, Seoul, Republic of Korea

**Keywords:** Brain tumor, Meningioma, Organoid, Precision medicine, Tumor microenvironment

## Abstract

**Background:**

Although meningioma is the most common primary brain tumor, treatments rely on surgery and radiotherapy, and recurrent meningiomas have no standard therapeutic options due to a lack of clinically relevant research models. Current meningioma cell lines or organoids cannot reflect biological features of patient tumors since they undergo transformation along culture and consist of only tumor cells without microenvironment. We aim to establish patient-derived meningioma organoids (MNOs) preserving diverse cell types representative of the tumor microenvironment.

**Methods:**

The biological features of MNOs were evaluated using WST, LDH, and collagen-based 3D invasion assays. Cellular identities in MNOs were confirmed by immunohistochemistry (IHC). Genetic alteration profiles of MNOs and their corresponding parental tumors were obtained by whole-exome sequencing.

**Results:**

MNOs were established from four patients with meningioma (two grade 1 and two grade 2) at a 100% succession rate. Exclusion of enzymatic dissociation-reaggregation steps endowed MNOs with original histology and tumor microenvironment. In addition, we used a liquid media culture system instead of embedding samples into Matrigel, resulting in an easy-to-handle, cost-efficient, and time-saving system. MNOs maintained their functionality and morphology after long-term culture (> 9 wk) and repeated cryopreserving-recovery cycles. The similarities between MNOs and their corresponding parental tumors were confirmed by both IHC and whole-exome sequencing. As a representative application, we utilized MNOs in drug screening, and mifepristone, an antagonist of progesterone receptor, showed prominent antitumor efficacy with respect to viability, invasiveness, and protein expression.

**Conclusion:**

Taken together, our MNO model overcame limitations of previous meningioma models and showed superior resemblance to parental tumors. Thus, our model could facilitate translational research identifying and selecting drugs for meningioma in the era of precision medicine.

**Supplementary Information:**

The online version contains supplementary material available at 10.1186/s12935-024-03225-4.

## Background

Meningioma is the most common primary brain tumor (comprising about 1/3 of all primary brain tumors) [1] and is sorted into three grades based on histopathological features according to World Health Organization (WHO) classification; Grade 1 (benign), 2 (atypical), and 3 (anaplastic) [2]. The treatment of meningioma is gross total resection with or without resection of dura mater, if indicated for surgery. Although benign meningiomas and completely resected meningiomas show relatively low recurrence rates (5–10%), atypical, anaplastic, or incompletely resected meningiomas due to anatomical location (e.g., skull base meningioma) show higher recurrence rates and require salvage radiotherapy [3, 4]. Until now, there have been no effective reagents for treating these patients, and clinical trials of several chemical agents have not significantly improved clinical outcomes [5, 6].

A lack of appropriate in vitro and in vivo models for meningioma is one of the reasons for the failure of development of systemic treatments [7]. As meningioma is a slowly growing tumor, establishment of tumor cells in 2D culture is more difficult than that of other malignant cancers. Although several meningioma cell lines have been established [8–10], additional genetic and phenotypic alterations acquired during long-term culture may hinder recapitulation of original tumors. Xenograft models in meningioma research are also limited due to the use of immunocompromised mice, eliminating the immune response within the tumor microenvironment. Xenograft models often rely on homogeneous cell lines, which do not reflect the true heterogeneity of human meningiomas [10, 11]. Additionally, establishment of in vivo mouse orthotopic xenograft model is difficult, time-consuming, and inefficient due to the unique anatomical location, originating from arachnoid cap cells [12]. Recently, patient-derived tumor organoids have gained the limelight as reliable and clinically relevant models. The organoid is a near-physiological model system representing characteristics of origin tissues, including molecular and histopathological features, cellular composition, and mutational profiles [13, 14]. Hence, it could be utilized as a pre-clinical model system for screening candidate therapeutic agents and predicting drug responses. Several tumor organoids have been established from diverse types of cancer including breast [15], lung [16], liver [17], pancreatic [18], and gastrointestinal cancers [19]. Patient-derived meningioma organoid (MNO) models were also reported recently [20, 21], but have limitations regarding reproducibility of the tumor microenvironment, partially owing to the enzymatic dissociation into single cells during tissue preparation.

In this study, we established a patient-derived organoid model from four patients with meningioma (two grade 1 and two grade 2) via an optimized protocol. Unlike previous MNO models [20, 21], our method can preserve the nature of tissue origin and tumor microenvironment, while minimizing preparation time. In addition, we confirmed that cryopreserving and recovery are possible for all MNOs without substantial alterations to their functions and phenotypes, indicating their potential applications in diverse in vitro experiments. As MNOs could be more efficiently established than xenograft models and more closely represent parental tumors than 2D culture, they could be utilized for precision medicine to treat meningioma.

## Methods

### Generation of MNOs from patient tissue

All tumor samples were pathologically diagnosed by a neuropathologist according to the 2021 WHO Classification of Tumors of the Central Nervous System, 5th edition. Fresh surgically resected meningioma tissue was obtained immediately after procedures at the Neuro-oncology Center of Seoul St. Mary’s Hospital and placed in sterile phosphate buffered saline. The generation of MNOs was performed as described in previous studies with some modifications [22, 23]. Briefly, the resected tissue was kept at 4 °C and placed in Hibernate A (BrainBits LLC, Springfield, IL, USA) supplemented with 1 × GlutaMax, 1 × PenStrep, and 1 × Amphotericin B, which were purchased from Thermo Fisher Scientific (Waltham, MA, USA), during tissue processing. Tumor tissue was mechanically minced into approximately 1 mm^3^-sized pieces using surgical scissors without enzymatic digestion. The tissue pieces were treated with RBC lysis buffer (Thermo Fisher Scientific), and then kept placed in growth medium on an orbital shaker rotating at 120 rpm in a 37 °C and 5% CO_2_ incubator throughout the maintenance. Growth medium is a mixture of 50% DMEM:F12 and 50% Neurobasal supplemented with 1 × GlutaMax, 1 × NEAAs, 1 × PenStrep, 1 × N2 supplement, 1 × B27 w/o vitamin A supplement, 1 × 2-mercaptoethnaol, and 2.5 μg/mL human insulin; human insulin solution was purchased from Sigma-Aldrich (St. Louis, MO, USA) and all others were obtained from Themo Fisher Scientific. The medium was changed twice a week and each MNO was cut when they get reached to diameters > 2 mm. For cryopreservation, MNOs were processed with 10 μM Y-27632 (Tocris Bioscience, Bristol, Avon, UK) for 1 h on orbital shaker and were placed with freezing medium containing growth medium with 10 μM Y-27632 and 10% DMSO (Sigma-Aldrich) in cryovials. Frozen MNOs were stored at − 80 °C in a deep freezer for 1 d, and then moved to liquid nitrogen tanks. For recovery, cryovials were quickly thawed in a water bath, and MNOs were placed in growth medium supplemented with 10 μM Y-27632 in a 37 °C and 5% CO_2_ incubator. The next day, MNOs were moved to normal growth medium on orbital shaker.

### Organoid viability and cytotoxicity assay

Single organoids were placed into individual wells of a 96-well culture plate with 100 μL of fresh medium per well. Growth of MNOs was measured by WST assays using Cell Counting Kit-8 (CCK-8) reagents (Dojindo Laboratories, Kumamoto, Japan). The plates were incubated with 10% CCK-8 reagent for 1.5 h. The absorbance was measured at 450 nm in a microplate reader (SYNERGY H1, Bio-Teck, Winooski, VT, USA). The quantification of plasma membrane damage and cytotoxicity was evaluated by lactate dehydrogenase (LDH) assay with LDH-Glo™ cytotoxicity Assay Kit (Promega Corporation, Fitchburg, WI, USA). Hydroxyurea (Sigma-Aldrich), everolimus (Sigma-Aldrich), mifepristone (Sigma-Aldrich), and temozolomide (Selleckchem, Houston, TX, USA) were used to test drug response in MNOs. Single organoids were transferred to 96-well plate with fresh medium and incubated for 72 h with drug treatment. After incubation, culture medium from each organoid was diluted 20-fold in PBS and then the diluted medium was mixed with LDH detection reagent at a 1:1 ratio. Luminescence was recorded after 1 h incubation at room temperature (RT). After that, samples were treated 0.2% Triton X-100 for 30 min to induce 100% cell death of MNOs, and cytotoxicity of each MNO was normalized to these positive controls.

### Histology and immunostaining

MNOs were fixed in 4% paraformaldehyde for 30 min–1 h, placed in a plastic cryomold, and snap frozen in tissue freezing medium on dry ice. Frozen blocks were stored at − 80 °C until processing. Sections (10–15 μm thick) of MNOs were sliced using a cryostat (Leica, Wetzlar, Hessen, Germany). Slides were dried at RT and stored at − 20 °C until ready for histologic evaluation. Hematoxylin–eosin (H&E) staining and immunohistochemistry (IHC) staining were performed following a widely used protocol described elsewhere [22, 23]. Masson’s trichrome staining was conducted to verify the collagenic nature in tumor tissues. In brief, tissue sections were deparaffinized and hydrated in distilled water, and the slides were then immersed in Bouin fluid with a subsequent cooling period. After rinsing, the sections were stained in Weigert’s hematoxylin, followed by Biebrich scarlet/acid fuchsin solution. The sections were then incubated in phosphomolybdic/phosphotungstic acid solution, dyed with aniline blue, and fixed with 1% acetic acid solution. Finally, the slides were thoroughly rinsed with distilled water. For H&E, Masson’s trichrome staining, and IHC using 3,3-diaminobenzidine (DAB) peroxidase (horseradish peroxidase, HRP), slides were scanned using a Pannoramic SCAN II (3DHISTECH Ltd, Budapest, Hungary) and representative images were captured by image viewing software (CaseViewer and Automated Slide Analysis Platform). For quantification of DAB staining, five to ten complete and non-overlapping regions of interest were randomly selected per each slide, and the percentage of positivity was then calculated using ImageJ. Images of immunofluorescence (IF) staining were captured using a Zeiss LSM 800 confocal microscope with 5 × and 40 × objective lens with Zen software (Zeiss, Jena, Thuringia, Germany). Information about antibodies used for immunostaining is shown in Additional file 1: Table S1.

### 3D invasion assay

Each organoid was embedded in matrix composed of type I collagen (Nitta Gelatin, Osaka, Japan) and Matrigel (Corning, NY, USA). The matrix mixture was prepared from collagen type I with Matrigel (1:1) in 2 × Ham’s F12 medium on ice. After that, the pH was adjusted by adding 10% reconstitution buffer (0.002 g/mL NaHCO_3_, 0.0047 g/mL HEPES, and 0.005 N NaOH). Matrix solution (100 μL) was directly pipetted into 96-well plates, and the single organoids were moved into the matrix prior to gelation. Culture medium was added over the gelled matrix to prevent the gel from drying out. The relative invasion area of each MNO was quantified by normalizing to the occupied area at 0 d.

### Whole-exome sequencing (WES)

DNA was extracted from approximately 15–20 MNOs (> 3 passages) and their parental tumor pieces (total 4 pairs, 8 samples). WES was performed using the Illumina NovaSeq platform (Illumina, San Diego, CA, USA) with an Agilent SureSelect V6-Post exome capture kit (Agilent Technologies Inc., Santa Clara, CA, USA). Burrows-Wheeler Aligner version 0.7.17 [24] was used to align raw sequencing data onto the GRCh38 human reference genome. Preprocessing of aligned data was performed under the somatic short variant discovery routine (single nucleotide variants, insertion, and deletion) in Best Practices Workflows of the genomic analysis tool kit (GATK) version 4.2.6.1 [25, 26]. Somatic mutations were called using the Mutect2 tumor-only mode. Annotation of variants was performed using ANNOVAR [27]. To filter variants, we removed those with allele frequencies ≥ 0.01 in the East Asian population of 1000 Genomes, ExAC, and the gnomAD_exome. Single nucleotide polymorphisms that have RS numbers in the dbSNP150 were also removed. Visualization of somatic variant information, including variant classification, was performed using maftools [28] in R. The similarities of copy number alteration patterns between MNOs and their parental tumors were assessed under the somatic copy number variant (CNV) discovery routine in Best Practices Workflows of the GATK. In DenoiseReadCounts and ModelSegments steps, each parental tumor was used as a reference copy number. The CallCopyRatioSegments was used as a segmentation algorithm to locate CNVs. Venn diagrams were drawn using the web-based tool InteractiVenn.

### Statistical analysis and software

Student’s *t*-test, one-way ANOVA, repeated measure ANOVA, Kruskal–Wallis test, and Shapiro–Wilk test were carried out using GraphPad Prism to calculate significance. Results are expressed as mean ± standard error of the mean (SEM).

## Results

### Establishment of patient-derived MNOs

**Fig. 1:**
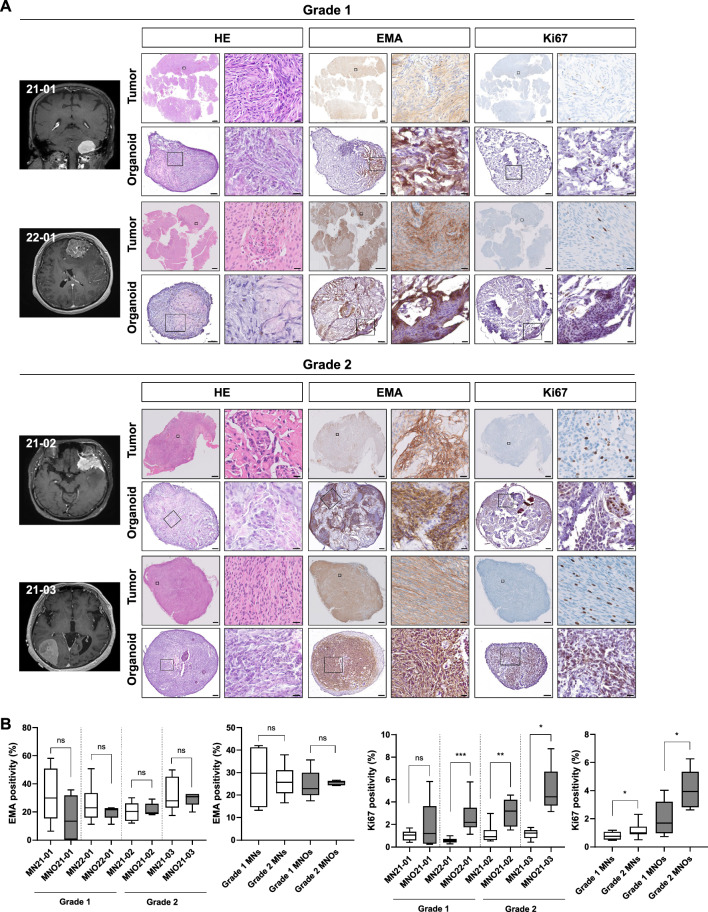
Radiographic and histological images of MNOs and their parental tumors. **A** Magnetic resonance images, H&E staining, and IHC using antibodies against EMA and Ki67 in MNOs and their parental tumors [grade 1 (21-01, 22-01) and grade 2 (21-02, 21-03)]. Scale bars indicate 2 mm (left) and 20 μm (right) in parental tumors, and 100 μm (left) and 20 μm (right) in organoids. **B** Quantification of the expression levels of EMA and Ki67. Two-tailed Student’s *t*-test was conducted to evaluate statistical significance (*P < 0.05, **P < 0.01, ***P < 0.001)

To establish patient-derived MNO models with high-reproducibility, we adopted the method reported to generate glioblastoma organoids [22, 23] with some modifications. Four surgically resected meningioma tumor tissues (Table 1) were mechanically minced without enzymatic digestion, and then cultured in growth medium on an orbital shaker throughout the maintenance (see Materials and Methods). Most MNOs developed spherical morphologies within 1–2 wk, and histological analyses showed consistencies with their corresponding parental tumors (Fig. 1a). All MNOs and their parental meningioma showed positive staining for EMA, a clinical diagnosis marker of meningiomas, with diffuse cytoplasmic patterns. Moreover, all four cases exhibited similar expression levels of EMA between MNOs and their parental tumors, and the difference between grade 1 and grade 2 was not significant (Fig. 1b). Notably, expression level of proliferation marker Ki67 was higher in MNOs derived from grade 2 meningioma than those from grade 1 meningioma, consistent with their parental tumors. These results coincide with the previous reports in which Ki67 positivity is increased according to tumor grade and risk of recurrence [29]. Furthermore, Ki67 expression was significantly higher in MNOs than corresponding tissues except the case 21–01 (Fig. 1b). We speculated that higher Ki-67 expressions in MNOs than those in parental tumors come from in vitro selection of rapidly proliferating cells during culture. Additionally, all four cases exhibited strong staining for Masson’s trichrome, a marker for collagen fibers (Additional file 1: Fig. S1).

**Table 1:** Clinical characteristics of patients with meningioma

Code	Sex	Age	Location	Diagnosis (grade)	Ki67	EMA	S-100	CD34	PR (allred score)
21-01	M	68	Cerebellum	Fibrous (1)	4%	+	−	−	4 (PS3 + IS1)
21-02	F	79	Sphenoid wing	Atypical (2)	8%	+	−	−	6 (PS3 + IS3)
21-03	F	75	Temporo-occipital	Atypical (2)	6%	+	−	+	8 (PS5 + IS3)
22-01	F	60	Frontal	Meningothelial (1)	2%	+	−	−	7 (PS5 + IS2)

PS, proportion score; IS, intensity score

**Fig. 2:**
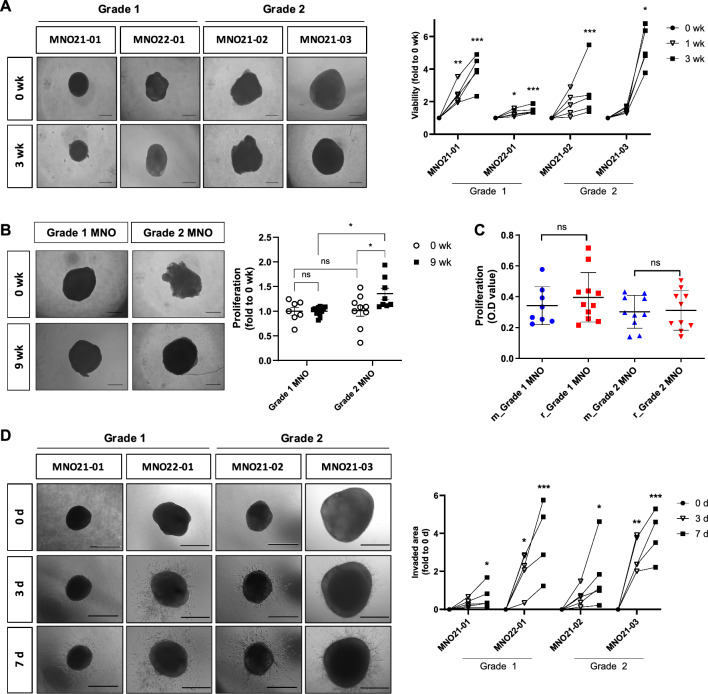
Functional characteristics of MNOs. **A** Bright-field microscopy images of individual growing MNOs. Quantification exhibits proliferation of individual MNOs and was measured by WST assay at each time point. Viability of MNOs was weekly measured by WST assays, and representative figures were captured by bright-field microscopy (Scale bar, 500 μm). Repeated measure ANOVA was performed to evaluate statistical significance compared with the 0 wk control (n = 5 per groups; *P < 0.05, **P < 0.01; ***P < 0.001). **B** Long-term culture of MNOs. Viability of MNOs were measured at each time point (0 and 9 wk) by WST assays, and representative figures were captured by bright-field microscopy (Scale bar, 500 μm). Each MNO was cut when they get reached to diameters > 2 mm and could not be applied to paired comparison due to long-term culture. One-way ANOVA with Tukey’s post hoc test was conducted to evaluate statistical significance (*P < 0.05). **C** Comparison of viabilities between continuously maintained MNOs (m_MNO) and recovered MNOs after cryopreservation (r_MNO). MNOs were recovered from the biobanks of two patients (grade 1: MNO21-01; grade 2: MNO21-02) (n = 8, m_MNO21-01; n = 11, r_MNO21-0; n = 10, m_MNO21-02; n = 10, r_MNO21-02). **D** Invasiveness of individual MNOs (n = 5) was measured at 3 d and 7 d using 3D invasion assays, and representative figures were captured by bright-field microscopy (Scale bar, 500 μm). Invaded areas were quantified using ToupView software. Repeated measure ANOVA was performed to evaluate statistical significance compared with the 0 d control (n = 5 per groups; *P < 0.05, **P < 0.01, ***P < 0.001). For **A**–**D**, Shapiro–Wilk test was performed to confirm normality of distribution

We next assessed biological phenotypes and functionalities of MNOs. Single MNOs were moved to each well of a 96-well plate, and their viabilities were measured weekly without cutting the MNOs. All four MNOs showed significantly increased viabilities until 3 wk, indicating gradual proliferation of MNOs (Fig. 2a). We also evaluated maintenance of MNO viability for long-term culture until 9 wk, and no substantial growth retardation was observed. In addition, grade 2 MNOs showed significantly higher proliferation rate than grade 1 MNOs after long-term culture, exhibiting significantly augmented proliferation rate than 0 wk MNOs (Fig. 2b). Banking-recovered MNOs exhibited similar growth rates to those continuously cultured without freezing–thawing cycles (Fig. 2c), suggesting that MNOs can be stocked like cancer cell lines. Additionally, we implanted MNOs in a collagen-Matrigel matrix to evaluate the invasive capacity of individual MNOs, and confirmed that all implanted MNOs definitely invaded the matrix (Fig. 2d). All these data suggest that MNOs preserve the nature of tissue origin and tumor microenvironment.

### Expression markers and cellular composition of MNOs

**Fig. 3:**
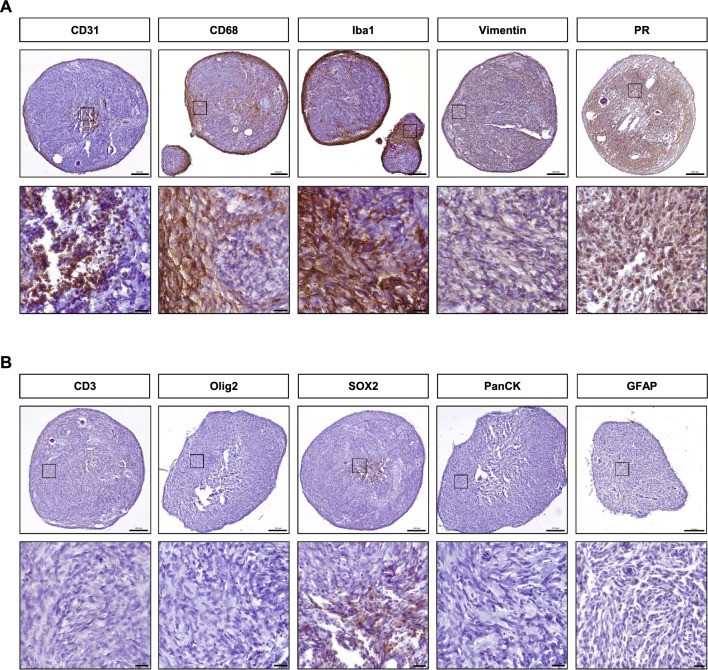
Expression of marker proteins in MNOs. **A** IHC images using antibodies against CD31, CD68, Iba1, Vimentin, and PR in MNO21-03. **B** IHC images using antibodies against CD3, Olig2, SOX2, PanCK, and GFAP in MNO21-03. Lower panels show the enlarged area marked by black boxes in the upper panels. Scale bars indicate 200 μm (upper panels) and 20 μm (lower panels)

To explore positive expression markers and further investigate cellular identities in MNOs, we performed immunohistochemical analyses using a variety of cell type markers, including CD31 for endothelial cells, CD68 and Iba1 for macrophage/microglia, CD3 for T cells, Olig2 and SOX2 for glioma stem cells, PanCK for epithelial cells, and GFAP for astrocytes. Additionally, Vimentin and progesterone receptor (PR), traditionally used for pathologic diagnosis [30, 31], were included. IHC showed positive expression of CD31, CD68, Iba1, Vimentin, and PR (Fig. 3a), whereas expression of Olig2, PanCK, and GFAP was not detected, and CD3 and SOX2 were weakly expressed (Fig. 3b). Expression of all these positive markers was recapitulated in IF staining, and the presence of macrophage/microglia and endothelial cells were confirmed by double staining of CD68 and Iba1, and CD31 and ICAM1, respectively (Additional file 1: Fig. S2). Notably, colocalized fluorescence showed the presence of macrophage/microglia along the rim of MNOs, coincident with previous findings where meningiomas with brain invasion show an immune response containing microglial/macrophagic cells at the tumor-brain border [32]. We also conducted DAB staining of CD68 and ICAM1 for a comparative analysis of the composition of macrophage/microglia and endothelial cells between MNOs and the corresponding parental tumors. All four cases exhibited similar expression levels of CD68 and ICAM1 between MNOs and their parental tumors (Additional file 1: Fig. S3). Taken together, these results demonstrate that MNOs were functionally well-established and contained specific cell types of the tumor microenvironment such as endothelial cells derived from blood vessel and immune cell populations.

### Recapitulation of genetic alterations between MNOs and parental tumors

**Fig. 4:**
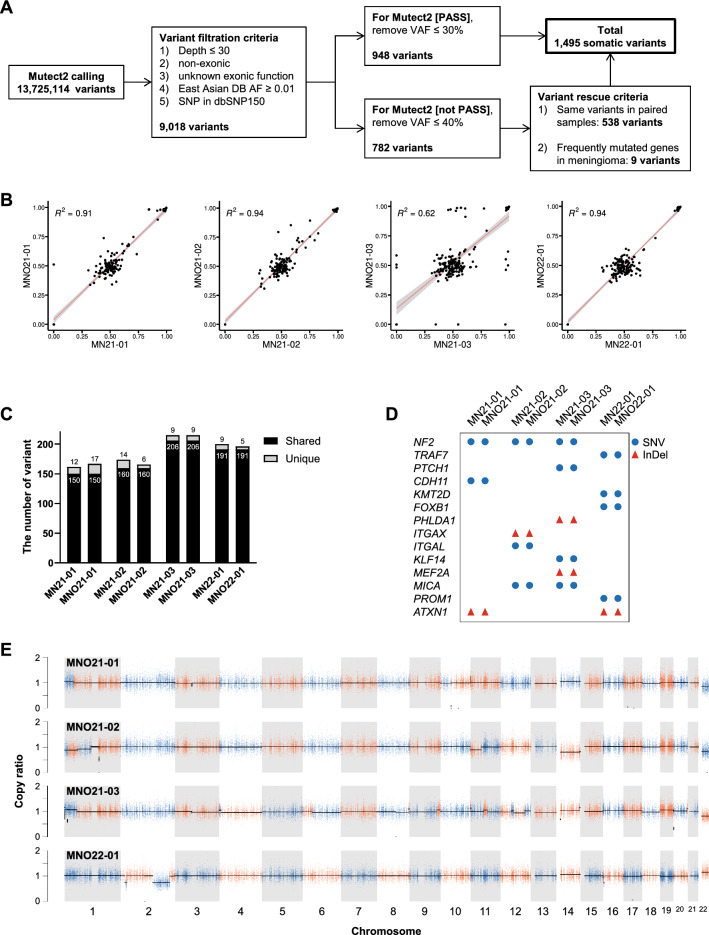
WES of MNOs and corresponding parental tumors. **A** Summary of variant calling and the refinement procedure. **B** Scatter plots representing variant allele frequencies of shared mutations between corresponding samples. Pearson’s correlation coefficients were displayed as R^2^. **C** The numbers of shared and unique variants between corresponding samples. **D** Variants in meningioma-associated genes identified by WES. The types of variants are displayed. **E** Copy number ratios of MNOs normalized to the corresponding parental tumors

To assess whether MNOs maintained genomic landscapes found in their corresponding parental tumors, we performed WES of four MNOs and their parental tumors (Fig. 4a and Additional file 1: Fig. S4A–C). Despite the heterogeneity among meningioma samples (Additional file 1: Fig. S4D, E), the majority of variants identified in parental tumors were found in corresponding MNOs at similar allele frequencies (Fig. 4b and Additional file 2), and most variants were shared between them (Fig. 4c). Notably, genetic alterations were not only reported to be frequently found in meningiomas (e.g., *NF2* and *TRAF7*) but also specifically found in each case (Fig. 4d). In addition, CNVs detected in parental tumors were also identified in corresponding MNOs (Fig. 4d). These results demonstrate that MNOs largely maintain the genetic profiles of corresponding parental tumors.

### MNOs as a drug screening model

**Fig. 5:**
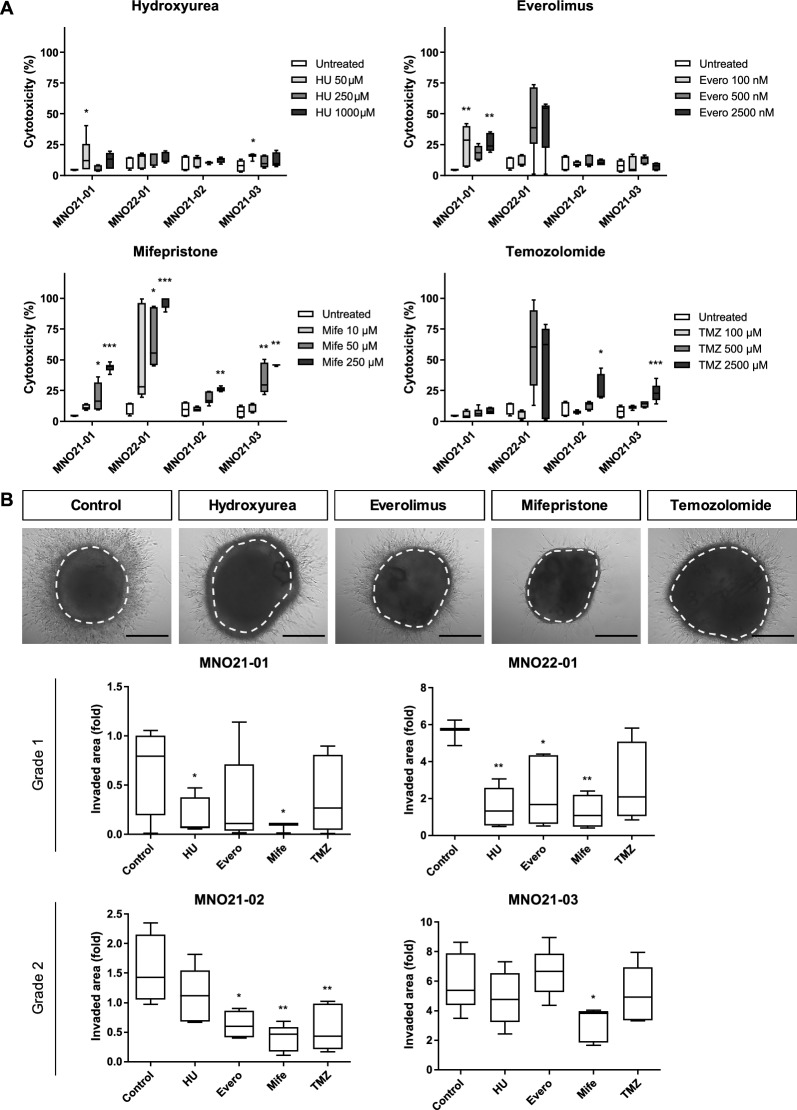
Screening of therapeutic reagents using MNOs. **A** Cytotoxicity was evaluated by LDH assays after treatment with four drugs for 72 h. Kruskal–Wallis test was performed with Dunn's post hoc correction (n = 6; *P < 0.05, **P < 0.01; ***P < 0.001). **B** 3D invasion assays after treatment with four drugs. Representative images were captured in MNO21-03 (Scale bar, 500 µm) and invasiveness was quantified at 7 d using ToupView software. One-way ANOVA was performed after confirmation of normality using Shapiro–Wilk test (*P < 0.05, **P < 0.01)

To explore the utility of MNOs as preclinical models for evaluation of drug responses, we tested four drugs based on their clinical relevance for meningioma treatment including standard-of-care therapies for other brain tumors and investigational drugs completed in clinical trials: hydroxyurea [33], everolimus [34], mifepristone [35], and temozolomide [36] using LDH cytotoxicity assays and 3D invasion assays (Fig. 5a, b). Interestingly, treatment with mifepristone, a competitive PR antagonist, exhibited dose-dependent cytotoxic responses and striking suppression of invasiveness in all MNOs (Fig. 5a, b). Temozolomide, one of the standard-of-care therapies in malignant glioma, also exhibited cytotoxic responses in a dose-dependent manner but could not suppress invasiveness of MNOs. Everolimus, an mTOR inhibitor, and hydroxyurea did not show consistent antitumor effects on MNOs.

**Fig. 6:**
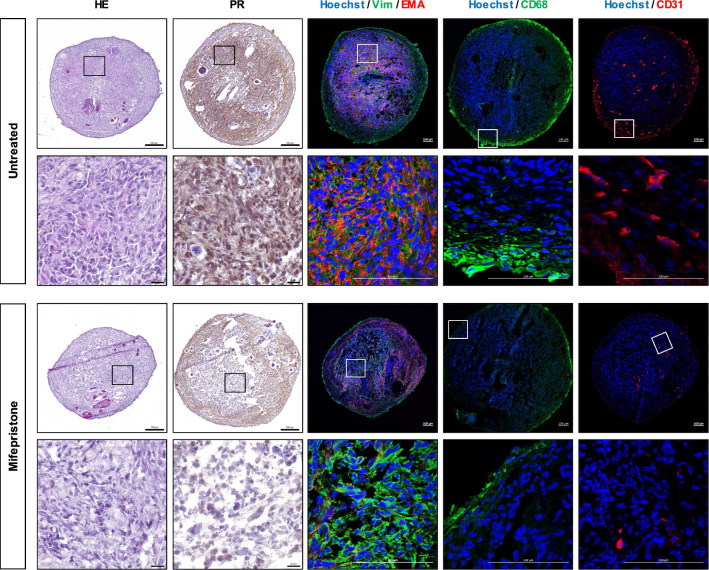
H&E and IHC images after treatment with mifepristone in MNO21-03. H&E and immunostaining (PR, EMA, Vimentin, CD68, and CD31) images were captured after treatment with mifepristone for 72 h. Lower panels show the enlarged areas marked by boxes in the upper panels. The nuclei were counterstained with Hoechst in IF images. Scale bars indicate 200 µm (top) and 20 µm (bottom) in H&E and DAB staining images, and indicate 100 µm (top and bottom) in IF images

**Fig. 7:**
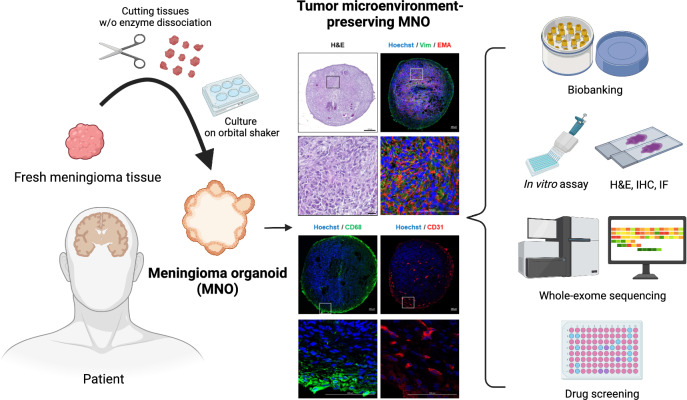
Schematic diagram of this study. A patient-derived MNO model, which includes diverse cell types of the tumor microenvironment and is practicable for cryopreserving-recovery cycles, was established. Biological and genetic features were validated using diverse in vitro assays and WES. Drug screening was performed as a representative application of MNO model

Owing to the remarkable efficacy of mifepristone, we further investigated the alteration of protein expression after treatment of MNOs with mifepristone. Expression of PR, EMA, CD68, and CD31 was considerably decreased in grade 2 MNOs by treatment with mifepristone (Fig. 6 and Additional file 1: Fig. S5). Grade 1 MNOs showed similar responses to mifepristone but those less prominent than grade 2 MNOs (Additional file 1: Figs. S6 and S7). These data suggest that mifepristone could suppress not only tumor cells originated from arachnoid cap cells, but also diverse cell types within tumor microenvironments such as endothelial cells and macrophages. The findings indicate that MNOs could be utilized as an in vitro drug testing platform to predict the treatment response of meningioma, particularly mifepristone. Figure 7 shows the schematic diagram of this study.

## Discussion

In vitro meningioma models representing individual patients are useful for investigating meningioma biology and preclinical drug testing. Here, we established patient-representing MNOs with high succession rate (100%), and revealed that they contain diverse cell types of the tumor microenvironment (Fig. 7). We also confirmed that the MNOs could maintain their biological features after long-term culture (9 wk) and cryopreservation-recovery cycles. The similarities between MNOs and parental tumors were assessed by both immunohistological features and genomic alteration profiles. As a representative application, we utilized MNOs in drug screening, and observed significant antitumor efficacy of mifepristone. Notably, antitumor efficacies of tested drugs varied among MNOs, which can explain the tumor heterogeneity and inconsistent drug responses among patients.

Numerous studies have recently reported methods of establishing diverse types of tumor organoids [37], but few previous studies explored the establishment of MNOs [20, 21], even with several limitations. In these previous models, MNOs were embedded into Matrigel, which is usually considered to trigger unknown adaptation of cells and subsequent phenotype alterations [38]. Moreover, they adopted enzymatic dissociation of patient tissues into single cells, so that organoids established by these methods cannot preserve the tumor microenvironment losing original composition and organization of parental tissues [16, 39]. Another previous study showed brain tumor organoids, which were directly derived from patient tissues, but they were not proved to preserve the tumor microenvironment or diverse stromal cells [40]. In this study, we dissected tumor tissues into small pieces without the enzymatic single-cell dissociation step, thereby our MNO model can maintain the tumor microenvironment with diverse cell types while reducing processing time. Endothelial cells, most likely derived from blood vessels, were distributed throughout MNOs, and macrophage/microglia cells surrounded the rims of MNOs (Figs. 3, 6). This feature can facilitate screening of candidate drugs targeting the tumor microenvironment such as anti-VEGF inhibitors and immunotherapy targeting macrophages [32, 39, 41]. We also used a liquid media culture system instead of embedding MNOs into Matrigel, resulting in an easy-to-handle, cost-efficient, and time-saving model. Notably, reduced processing time due to our optimized protocol might increase the succession rate of organoid culture because the viability of cells in fresh tissue is more vulnerable than stabilized and isolated contexts [42]. Although we used simplified protocols, cryopreservation and recovery cycles did not retard proliferation of MNOs, indicating their value as a research model system.

Patient-derived organoids can be predictive of patient’s therapeutic responses and guide clinical treatment to improve prognosis [43]. Using MNOs, we revealed that the PR inhibitor mifepristone has remarkable antitumor efficacies against meningioma, consistent with recent reports associating the prognostic significance of elevated PR expression in meningiomas [44]. This is supported by several preclinical and clinical studies, which demonstrated that mifepristone was effective in treating unresectable meningiomas, potentially due to its ability to block PR [45, 46]. The anti-proliferative effects of mifepristone in meningiomas could also be attributed to its ability to induce apoptosis, as suggested by studies on other types of cancer cells [47]. Furthermore, mifepristone has been shown to have anti-angiogenic properties, which could contribute to its antitumor effects by disrupting the blood supply to the tumor [48]. Based on alterations of marker expression in MNOs (Fig. 6), we inferred that mifepristone hampers microvascular formation and immune response in meningioma, resulting in suppression of aggressive behavior of meningioma. Since a greater number of MNOs are required to strengthen our hypothesis, we will further establish MNOs from each patient with diverse clinical information. Validation through additional large cohort studies could facilitate the identification of potential biomarkers that could serve as predictors of individual responsiveness to mifepristone. In addition, we will analyze single cell transcriptional program of MNOs in the presence or absence of specific drugs to precisely elucidate detailed action mechanisms of drugs and further explore effects of diverse tumor microenvironmental cells.

## Conclusion

Our study demonstrates establishment of biobanking-recovery available and tumor microenvironment-preserving organoid model from patients with meningioma. Until now, stabilizing meningioma models in vitro or in vivo are rare due to its less aggressive nature, but our MNO model overcame these limitations and is superior to others in terms of resemblance to parental tumors. We dissected tumor tissues into small pieces without the enzymatic single-cell dissociation step, thereby our MNO model can maintain the tumor microenvironment with diverse cell types while reducing processing time. In particular, WES results showed that the majority of variants was shared between MNOs and their parental tumors with few unique variants. Thus, we expect that our method can facilitate preclinical screening and selecting drugs for meningioma. In future work, we will try to identify novel drugs for meningioma based on the WES data of parental tumors and therapeutic responses of corresponding MNOs achieving precision medicine.

### Supplementary Information

**Additional file 1. Fig. S1**: Masson’s trichrome staining in MNOs and their parental tumors. Collagen-rich tissue (blue) was observed using Masson’s trichrome in MNOs (top) and their corresponding tumors (bottom). Blue = collagen fiber; red/pink = cytoplasm; dark red/purple = nuclei. Scale bars: upper images = 200 μm; lower images = 20 μm. Glioblastoma organoid (GBO) was used as a negative control. **Fig. S2**: IF images of marker proteins in MNOs. IF images using antibodies against EMA, Vimentin, CD31, ICAM1, CD68, and Iba1 in all MNOs. Lower panels show the enlarged areas marked by boxes in the upper panels. The nuclei were counterstained with Hoechst. White scale bars = 100 μm; yellow scale bars = 20 μm. **Fig. S3**: DAB staining images for CD68 and ICAM1 in MNOs and their parental tumors. **A**, **B** DAB staining for CD68 (**A**) and ICAM1 (**B**) in MNOs (lower panel) and parental meningioma tissues (upper panel). Scale bars indicate 200 μm (top) and 20 μm (bottom, enlarged images). **C** Quantification of the expression levels of CD68 (left) and ICAM1 (right). Two-tailed Student’s *t*-test was conducted to evaluate statistical significance. **Fig. S4**: WES of MNOs and corresponding parental tumors. **A**, **B** The number of variants and their types for each sample. **C** The number of each class of single nucleotide variants. **D**, **E** Venn diagram of the number of short variants (single nucleotide variants, insertions, and deletions) for parental tumors (**D**) and MNOs (**E**). **Fig. S5**: H&E and IHC images after treatment with mifepristone in MNO21-02. H&E and immunostaining (PR, EMA, Vimentin, CD68, and CD31) images were captured after treatment with mifepristone for 72 h. Lower panels show the enlarged areas marked by boxes in the upper panels. The nuclei were counterstained with Hoechst in IF images. Scale bars indicate 200 μm (top) and 20 μm (bottom) in H&E and DAB staining images, and indicate 100 μm (top and bottom) in IF images. **Fig. S6**: H&E and IHC images after treatment with mifepristone in MNO21-01. H&E and immunostaining (PR, EMA, Vimentin, CD68, and CD31) images were captured after treatment with mifepristone for 72 h. Lower panels show the enlarged areas marked by boxes in the upper panels. The nuclei were counterstained with Hoechst in IF images. Scale bars indicate 200 μm (top) and 20 μm (bottom) in H&E and DAB staining images, and indicate 100 μm (top and bottom) in IF images. **Fig. S7**: H&E and IHC images after treatment with mifepristone in MNO22-01. H&E and immunostaining (PR, EMA, Vimentin, CD68, and CD31) images were captured after treatment with mifepristone for 72 h. Lower panels show the enlarged areas marked by boxes in the upper panels. The nuclei were counterstained with Hoechst in IF images. Scale bars indicate 200 μm (top) and 20 μm (bottom) in H&E and DAB staining images, and indicate 100 μm (top and bottom) in IF images. **Table S1**: The information of antibodies used for immunostaining.

Additional file 2. **Table S1**. The number of somatic mutations obatined from WES data. **Table S2**. List of identified somatic mutations in MN21-01. **Table S3**. List of identified somatic mutations in MNO21-01. **Table S4**. List of identified somatic mutations in MN21-02. **Table S5**. List of identified somatic mutations in MNO21-02. **Table S6**. List of identified somatic mutations in MN21-03. **Table S7**. List of identified somatic mutations in MNO21-03. **Table S8**. List of identified somatic mutations in MN22-01. **Table S9**. List of identified somatic mutations in MNO22-01.

## Data Availability

The datasets generated and/or analyzed during the current study are available in the NCBI SRA database under Accession No. PRJNA882595.

## Abbreviations

CNV: Copy number variant
DAB: 3,3-Diaminobenzidine
GATK: Genomic analysis tool kit
H&E: Hematoxylin–eosin
HRP: Horseradish peroxidase
IF: Immunofluorescence
IHC: Immunohistochemistry
LDH: Lactate dehydrogenase
MNO: Meningioma organoid
PBS: Phosphate-buffered saline
PR: Progesterone receptor
TBS: Tris-buffered saline
WES: Whole-exome sequencing
WHO: World Health Organization
