# Supplementary material for: Establishment of tumor microenvironment-preserving organoid model from patients with intracranial meningioma
Source: Cancer Cell Int. 2024 Jan 18;24:36. doi: 10.1186/s12935-024-03225-4 (PMC10795458; doi:10.1186/s12935-024-03225-4)
Supplement: Supplementary file 1 — Additional file 1. Fig. S1: Masson’s trichrome staining in MNOs and their parental tumors. Collagen-rich tissue (blue) was observed using Masson’s trichrome in MNOs (top) and their corresponding tumors (bottom). Blue = collagen fiber; red/pink = cytoplasm; dark red/purple = nuclei. Scale bars: upper images = 200 μm; lower images = 20 μm. Glioblastoma organoid (GBO) was used as a negative control. Fig. S2: IF images of marker proteins in MNOs. IF images using antibodies against EMA, Vimentin, CD31, ICAM1, CD68, and Iba1 in all MNOs. Lower panels show the enlarged areas marked by boxes in the upper panels. The nuclei were counterstained with Hoechst. White scale bars = 100 μm; yellow scale bars = 20 μm. Fig. S3: DAB staining images for CD68 and ICAM1 in MNOs and their parental tumors. A, B DAB staining for CD68 (A) and ICAM1 (B) in MNOs (lower panel) and parental meningioma tissues (upper panel). Scale bars indicate 200 μm (top) and 20 μm (bottom, enlarged images). C Quantification of the expression levels of CD68 (left) and ICAM1 (right). Two-tailed Student’s t-test was conducted to evaluate statistical significance. Fig. S4: WES of MNOs and corresponding parental tumors. A, B The number of variants and their types for each sample. C The number of each class of single nucleotide variants. D, E Venn diagram of the number of short variants (single nucleotide variants, insertions, and deletions) for parental tumors (D) and MNOs (E). Fig. S5: H&E and IHC images after treatment with mifepristone in MNO21-02. H&E and immunostaining (PR, EMA, Vimentin, CD68, and CD31) images were captured after treatment with mifepristone for 72 h. Lower panels show the enlarged areas marked by boxes in the upper panels. The nuclei were counterstained with Hoechst in IF images. Scale bars indicate 200 μm (top) and 20 μm (bottom) in H&E and DAB staining images, and indicate 100 μm (top and bottom) in IF images. Fig. S6: H&E and IHC images after treatment with mifepristone in MNO21-01. H&E an [file 12935_2024_3225_MOESM1_ESM.pdf]

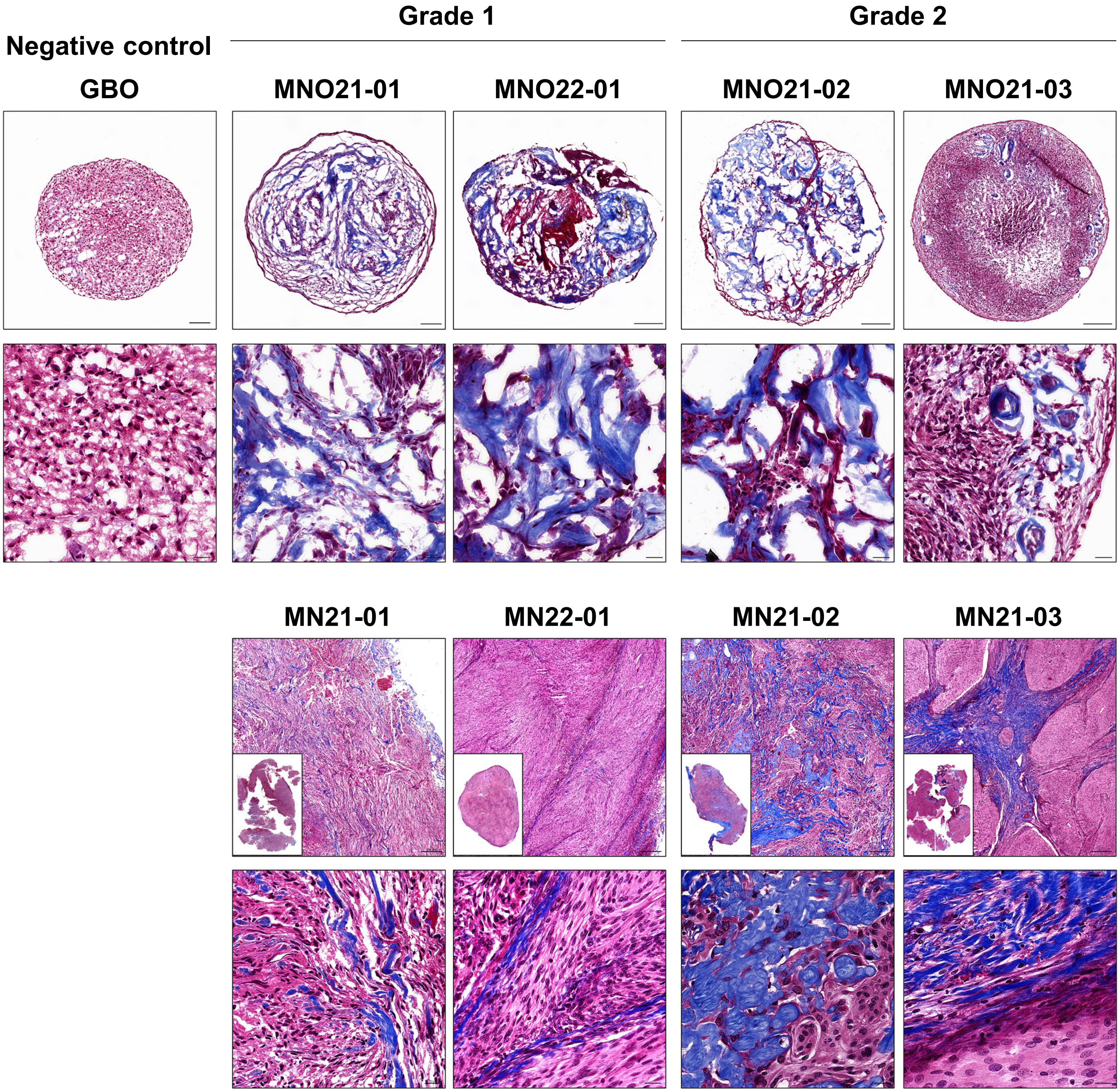

Fig. S1 Masson's trichrome staining in MNOs and their parental tumors. Collagen-rich tissue (blue) was observed using Masson's trichrome in MNOs (top) and their corresponding tumors (bottom). Blue = collagen fiber; red/pink = cytoplasm; dark red/purple = nuclei. Scale bars: upper images = 200 μm; lower images = 20 μm. Glioblastoma organoid (GBO) was used as a negative control.

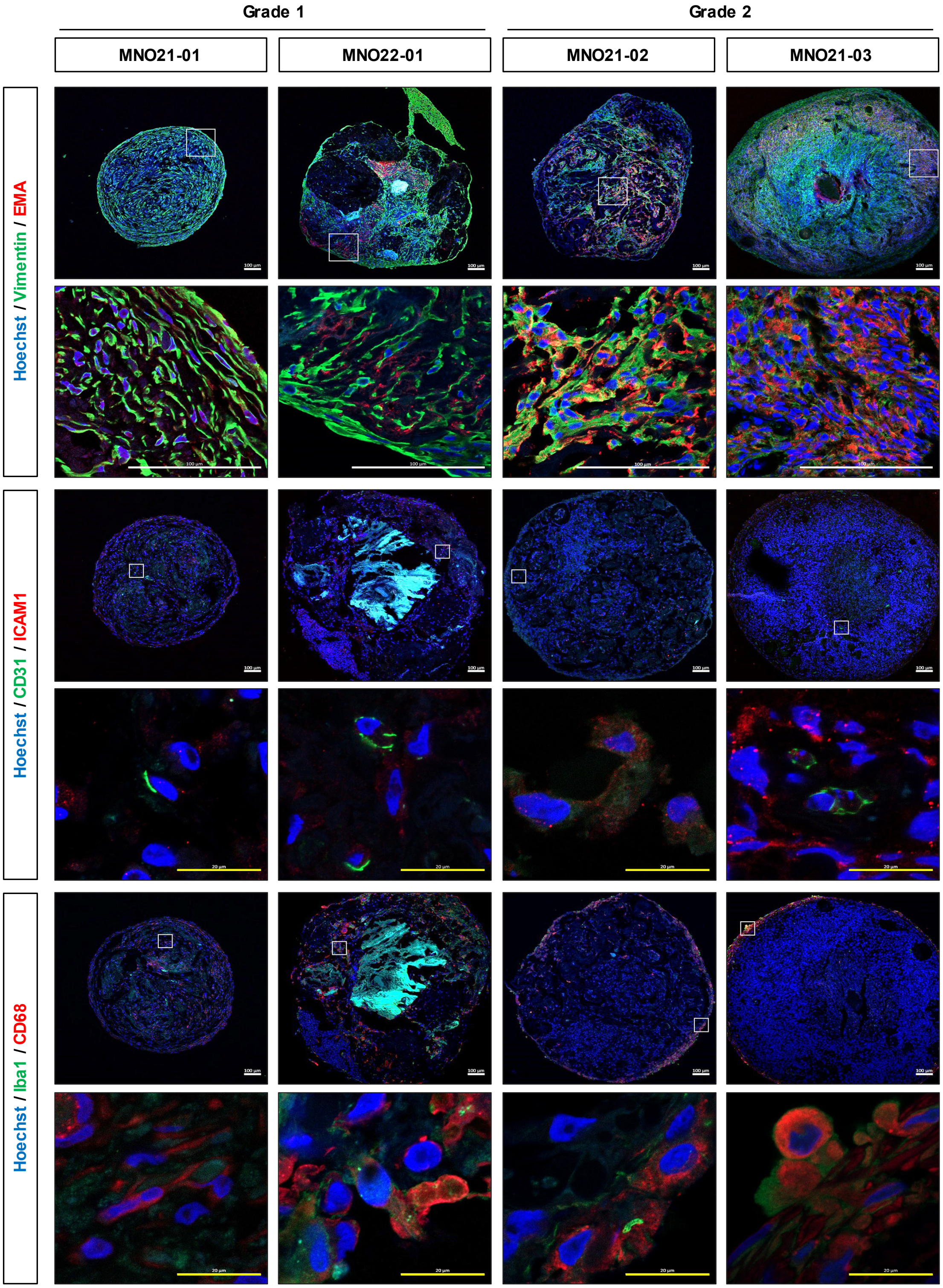

Fig. S2 IF images of marker proteins in MNOs. IF images using antibodies against EMA, Vimentin, CD31, ICAM1, CD68, and Iba1 in all MNOs. Lower panels show the enlarged areas marked by boxes in the upper panels. The nuclei were counterstained with Hoechst. White scale bars = 100 µm; yellow scale bars = 20 µm.

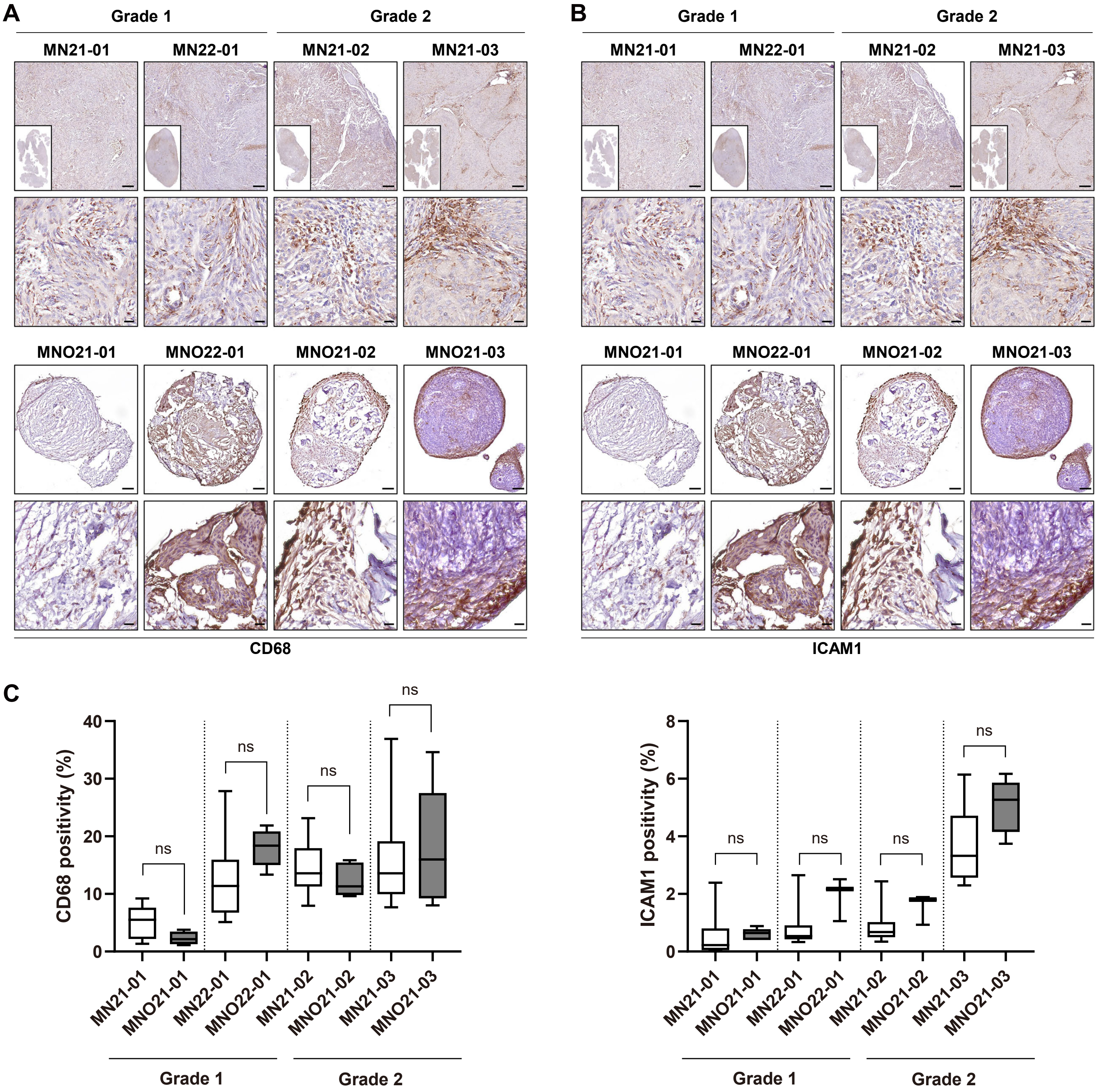

Fig. S3 DAB staining images for CD68 and ICAM1 in MNOs and their parental tumors. (**A** and **B**) DAB staining for CD68 (**A**) and ICAM1 (**B**) in MNOs (lower panel) and parental meningioma tissues (upper panel). Scale bars indicate 200 µm (top) and 20 µm (bottom, enlarged images). (**C**) Quantification of the expression levels of CD68 (left) and ICAM1 (right). Two-tailed Student's *t*-test was conducted to evaluate statistical significance.

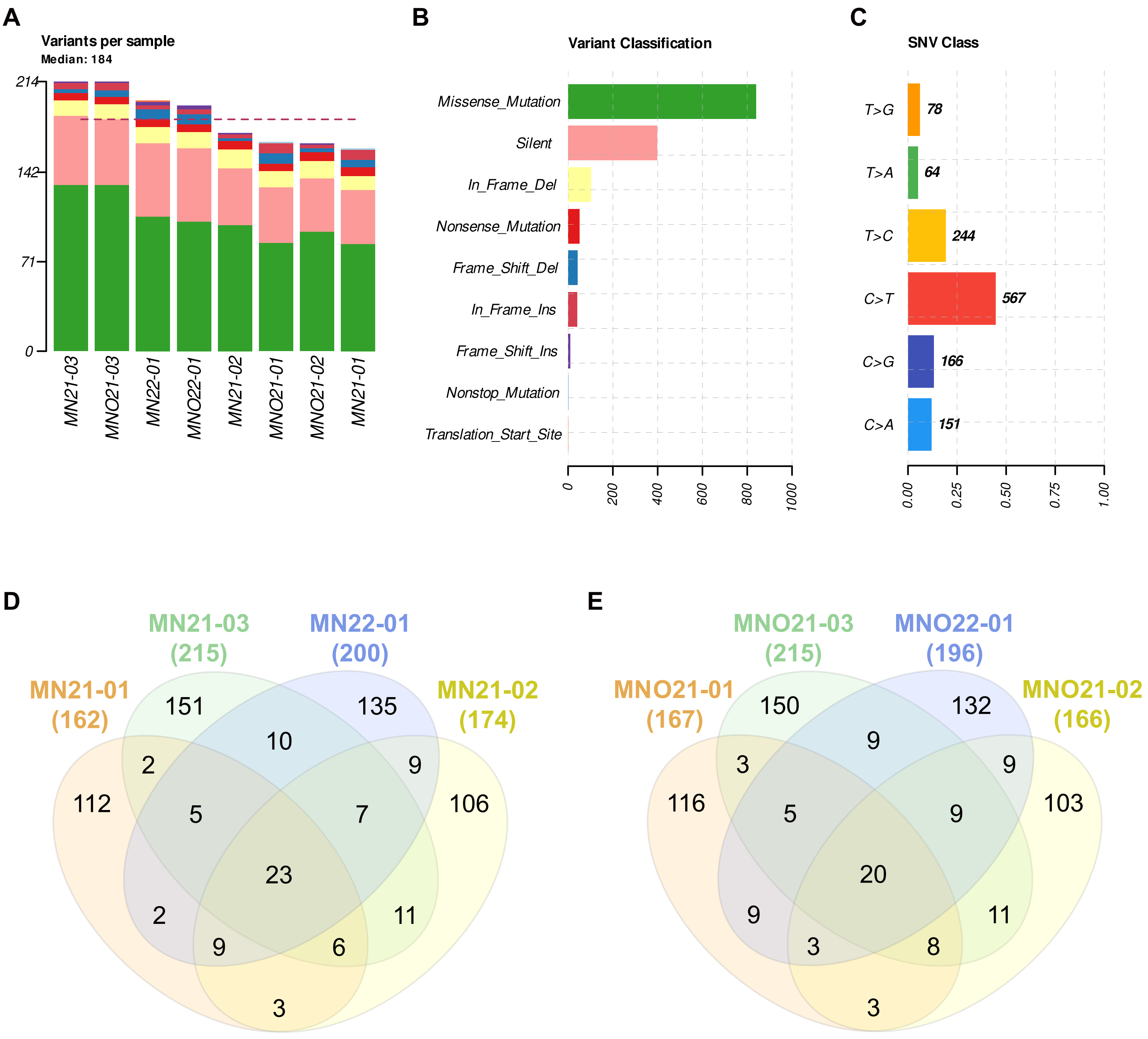

Fig. S4 WES of MNOs and corresponding parental tumors. (**A** and **B**) The number of variants and their types for each sample. (**C**) The number of each class of single nucleotide variants. (**D** and **E**) Venn diagram of the number of short variants (single nucleotide variants, insertions, and deletions) for parental tumors (**D**) and MNOs (**E**).

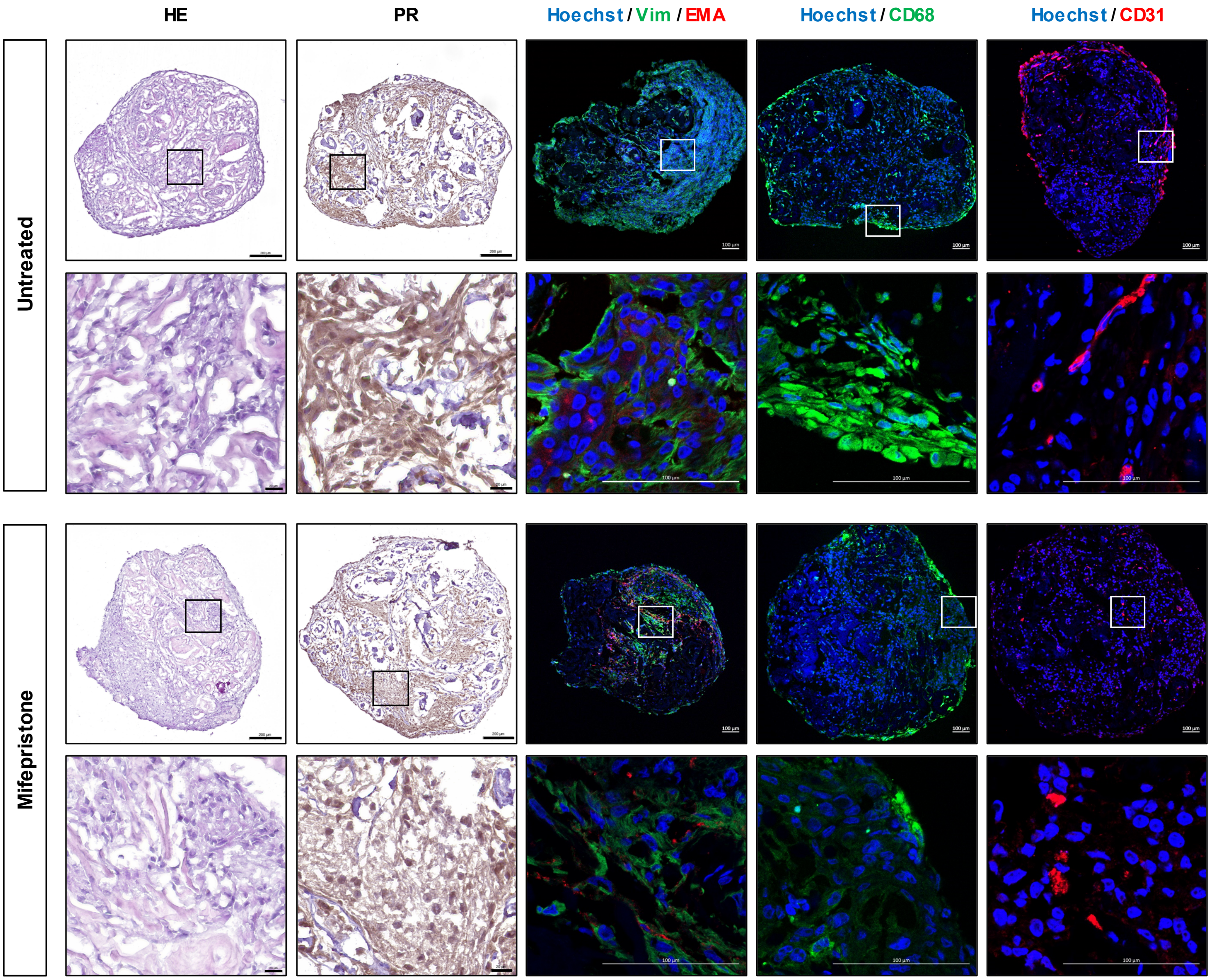

Fig. S5 H&E and IHC images after treatment with mifepristone in MNO21-02. H&E and immunostaining (PR, EMA, Vimentin, CD68, and CD31) images were captured after treatment with mifepristone for 72 h. Lower panels show the enlarged areas marked by boxes in the upper panels. The nuclei were counterstained with Hoechst in IF images. Scale bars indicate 200 µm (top) and 20 µm (bottom) in H&E and DAB staining images, and indicate 100 µm (top and bottom) in IF images.

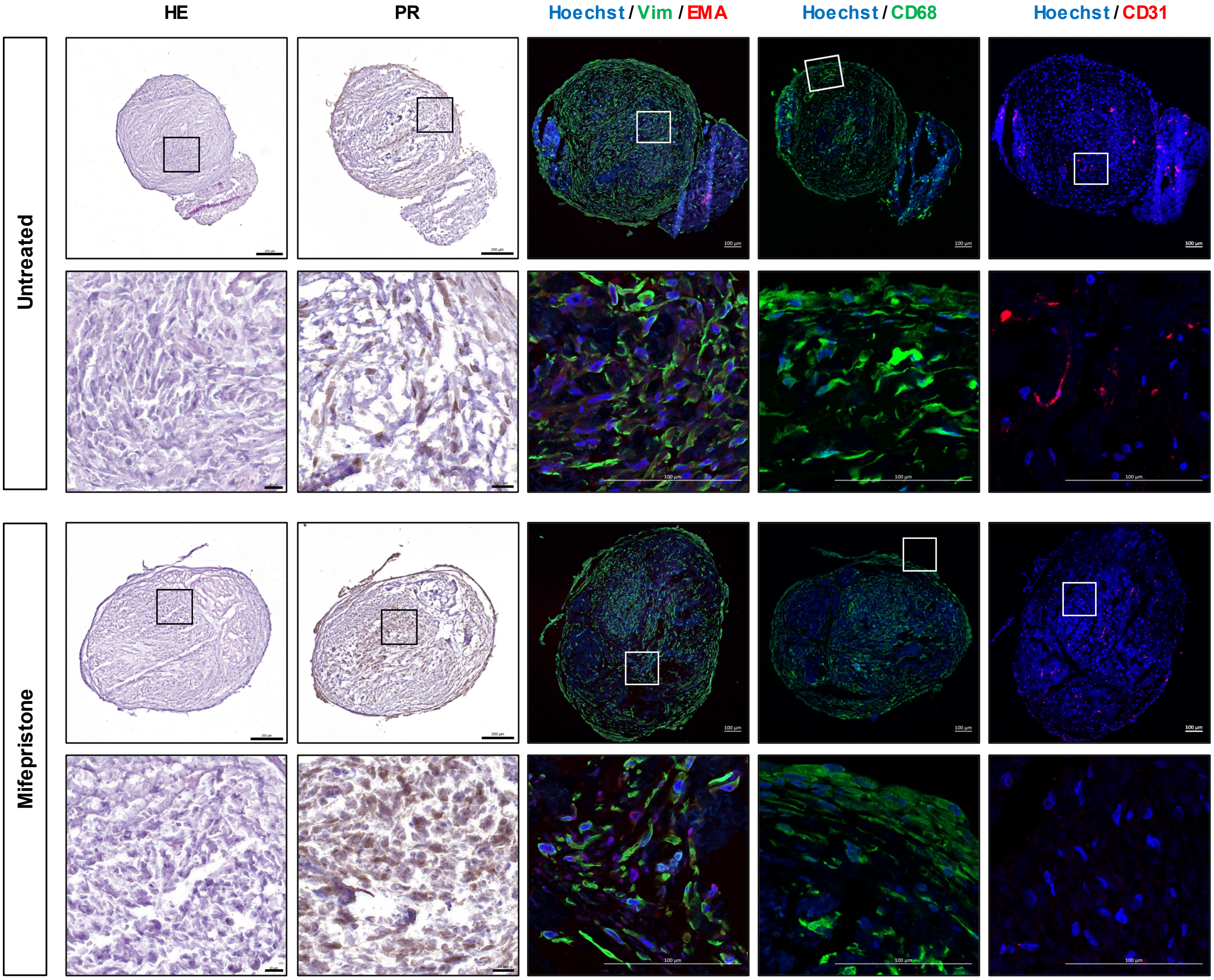

Fig. S6 H&E and IHC images after treatment with mifepristone in MNO21-01. H&E and immunostaining (PR, EMA, Vimentin, CD68, and CD31) images were captured after treatment with mifepristone for 72 h. Lower panels show the enlarged areas marked by boxes in the upper panels. The nuclei were counterstained with Hoechst in IF images. Scale bars indicate 200 µm (top) and 20 µm (bottom) in H&E and DAB staining images, and indicate 100 µm (top and bottom) in IF images.

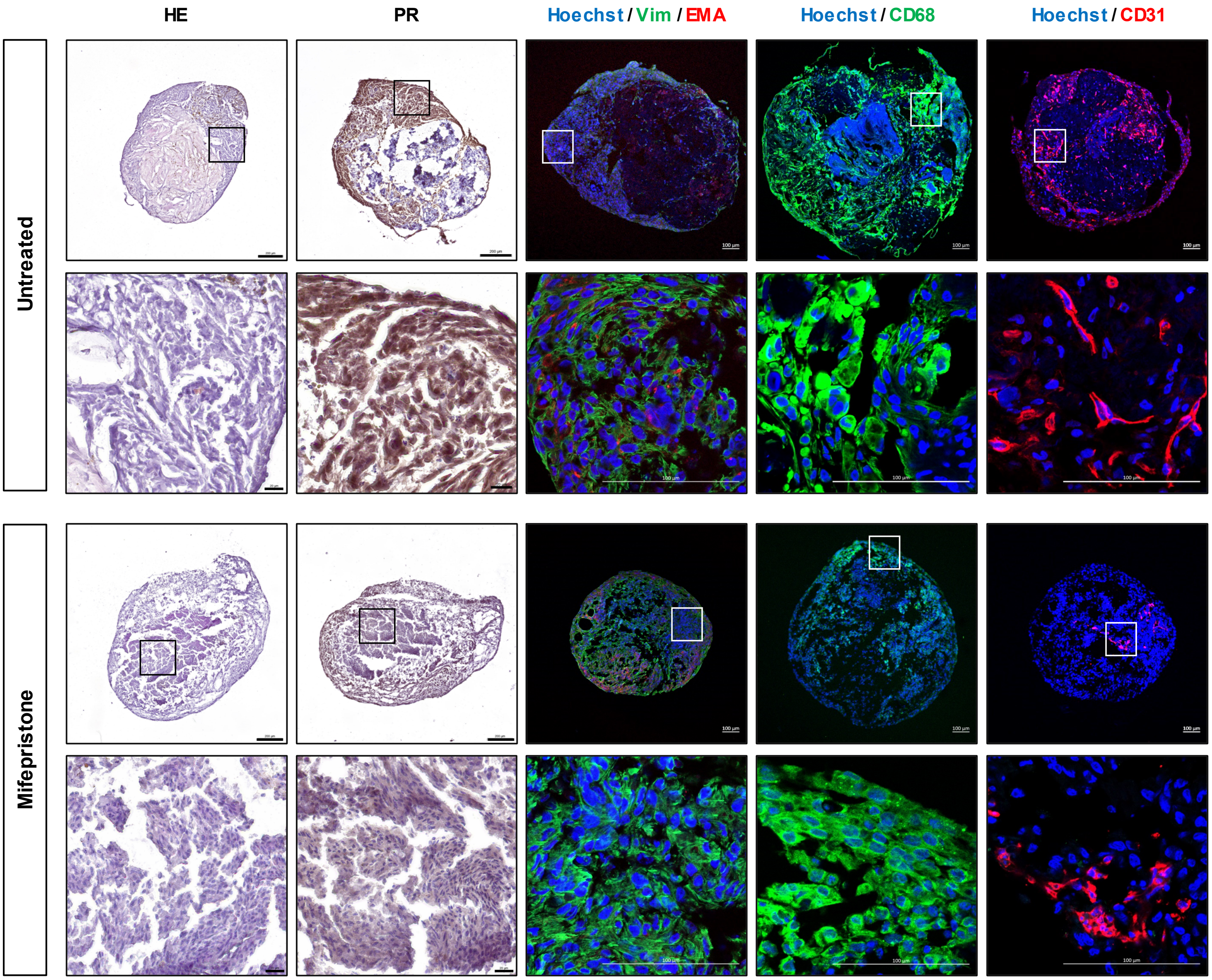

Fig. S7 H&E and IHC images after treatment with mifepristone in MNO22-01. H&E and immunostaining (PR, EMA, Vimentin, CD68, and CD31) images were captured after treatment with mifepristone for 72 h. Lower panels show the enlarged areas marked by boxes in the upper panels. The nuclei were counterstained with Hoechst in IF images. Scale bars indicate 200 µm (top) and 20 µm (bottom) in H&E and DAB staining images, and indicate 100 µm (top and bottom) in IF images.

Table S1 The information of antibodies used for immunostaining.

Antibodies	Dilution	Source	Catalog
Mouse monoclonal anti-Ki67	1:500	Abcam	ab245113
Rabbit polyclonal anti-Ki67	1:200	Abcam	ab15580
Rabbit monoclonal anti-Vimentin	1:800	Cell Signaling Technology	5741
Mouse monoclonal anti-CD31	1:500	Abcam	ab9498
Rabbit monoclonal anti-CD3	1:300	Abcam	ab16669
Mouse monoclonal anti-GFAP	1:700	Cell Signaling Technology	3670
Rabbit monoclonal anti-Olig2	1:300	Abcam	ab109186
Rabbit polyclonal anti-SOX2	1:1000	Abcam	ab97959
Mouse monoclonal anti-Epithelial Membrane Antigen (EMA)	1:250	Agilent Technologies	M0613
Mouse monoclonal anti-CD68	1:100	Abcam	ab955
Mouse monoclonal Anti-pan Cytokeratin (panCK)	1:500	Abcam	ab7753
Rabbit monoclonal Anti-Iba1	1:500	Abcam	ab178846
Mouse monoclonal anti-Progesterone receptor (PR)	1:500	Santa Cruz Biotechnology	sc-166169
Dako REAL EnVision Detection System, Perox/DAB+, Rb/M	N/A	Agilent Technologies	K500711-2
Goat anti-rabbit IgG-FITC	1:1000	Santa Cruz Biotechnology	sc-2012
Goat anti-mouse IgG-CFL 647	1:500	Santa Cruz Biotechnology	sc-362287
